# Quantification of huntingtin protein species in Huntington’s disease patient leukocytes using optimised electrochemiluminescence immunoassays

**DOI:** 10.1371/journal.pone.0189891

**Published:** 2017-12-22

**Authors:** Davina J. Hensman Moss, Nicola Robertson, Ruth Farmer, Rachael I. Scahill, Salman Haider, Michela A. Tessari, Geraldine Flynn, David F. Fischer, Edward J. Wild, Douglas Macdonald, Sarah J. Tabrizi

**Affiliations:** 1 Huntington’s Disease Centre, Department of Neurodegenerative Disease, UCL Institute of Neurology, London, United Kingdom; 2 Department of Medical Statistics, London School of Hygiene and Tropical Medicine, London, United Kingdom; 3 Galapagos B. V., Leiden, Netherlands; 4 Charles River, Leiden, Netherlands; 5 CHDI Management/CHDI Foundation, Los Angeles, California, United States of America; University of Florida, UNITED STATES

## Abstract

**Background:**

Huntington’s disease (HD) is an autosomal dominant neurodegenerative condition caused by an expanded CAG repeat in the gene encoding huntingtin (HTT). Optimizing peripheral quantification of huntingtin throughout the course of HD is valuable not only to illuminate the natural history and pathogenesis of disease, but also to detect peripheral effects of drugs in clinical trial.

**Rationale:**

We previously demonstrated that mutant HTT (mHTT) was significantly elevated in purified HD patient leukocytes compared with controls and that these levels track disease progression. Our present study investigates whether the same result can be achieved with a simpler and more scalable collection technique that is more suitable for clinical trials.

**Methods:**

We collected whole blood at 133 patient visits in two sample sets and generated peripheral blood mononuclear cells (PBMCs). Levels of mHTT, as well as N-, and C-terminal and mid-region huntingtin were measured in the PBMCs using ELISA-based Meso Scale Discovery (MSD) electrochemiluminescence immunoassay platforms, and we evaluated the relationship between different HTT species, disease stage, and brain atrophy on magnetic resonance imaging.

**Conclusions:**

The assays were sensitive and accurate. We confirm our previous findings that mHTT increases with advancing disease stage in patient PBMCs, this time using a simple collection protocol and scalable assay.

## Introduction

Huntington’s disease is a devastating neurodegenerative disease caused by a CAG repeat expansion in exon 1 of the *HTT* gene, encoding an expanded polyglutamine in the ubiquitously-expressed HTT protein. Mutant HTT (mHTT) expression is the primary pathogenic factor for the development of HD, with increasing expression levels associated with disease severity and toxicity in various models [1–3]. HD is autosomal dominant and fully penetrant, which, combined with the availability of a genetic test, makes the disease highly tractable [4–8]; however, biofluid biomarkers are limited [9]. There are currently no disease-modifying therapies for HD but putative therapeutic approaches aim to lower mHTT levels in the CNS [3], with the first trial of a HTT-lowering drug entering Phase 1/2a trial in 2015 [10]. Peripheral biomarkers would further improve the understanding of HD natural history, and could be sensitive to peripherally-administered therapies. Thus though quantification of mutant and wild-type Huntingtin and their cleaved or truncated species in living Huntington’s disease (HD) patients is challenging, it remains a desirable objective.

Blood is readily available, and since peripheral immune system dysfunction is a feature of HD and tracks disease progression [11–15], blood-based biomarkers are an area of interest. We previously used a time-resolved Förster resonance energy transfer (TR-FRET) immunoassay to demonstrate that mHTT was significantly elevated in purified HD patient monocytes and lymphocytes compared to controls, increased progressively with advancing disease stage, and was associated with both disease burden score and caudate atrophy rate [13]. A disadvantage of this technique is its dependence on FRET distance, which creates an unpredictable relationship between CAG repeat length, protein concentration and FRET signal. Furthermore, the initial processing required to obtain purified leukocyte subpopulations is relatively complicated, making it challenging to apply to multi-site clinical trial settings. We recently described assays that measure either polyglutamine-independent human HTT or polyglutamine-expanded human HTT proteins on the electrochemiluminescence Meso Scale Discovery (MSD) detection platform, and demonstrated that these assays are sensitive and selective in model systems [16]. This MSD platform enables multiple states or species of HTT to be measured in complex tissues and fluids by using epitope-directed antibodies, and is more suited to high-throughput studies.

**Fig 1:**
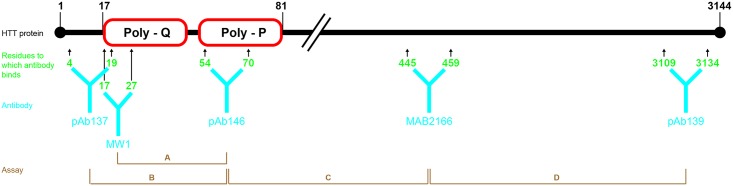
Schematic diagram showing the capture and detection antibody pairs for each assay. The huntingtin amino acid residue epitope to which the antibodies bind are shown (not to scale). Poly-Q, polyglutamine region; Poly-P, proline rich region. Details of the antibodies are given in the text below.

Our work here explores whether the mHTT trends associations described above which were detected in leukocyte subpopulations [13] could also be detected sensitively and accurately for the first time in mixed leukocytes from HD patient samples using the MSD assay system. We used a simple blood collection protocol that could be readily used at multiple study sites as part of large-scale clinical trials: sampling tubes allow isolation of peripheral blood mononuclear cells (PBMCs, including lymphocytes and monocytes) using a single-step process [17–19]. Using the MSD platform assays we used different antibody combinations to detect (1) polyglutamine-expanded (mutant) N-terminal HTT, (2) polyglutamine-independent HTT N-terminus, (3) polyglutamine-independent HTT mid-region, and (4) polyglutamine-independent HTT C-terminus of HTT [16] (Fig 1). We then characterized the relationship between HTT species, disease stage, and brain imaging variables.

## Materials and methods

### Study design and subject selection

Two cross-sectional studies were conducted nine months apart with a total of 133 visits from 101 participants (32 subjects were assessed on both visits) (Table 1 and S1 Table).

**Table 1:** Demographic details of sample sets used.

	Sample set 1	Sample set 2	Number of subjects repeated
**Control**	20 (11)	23 (14)	13 (8)
**Premanifest HD**	14 (7)	23 (14)	7 (5)
**Early HD**	14 (7)	18 (7)	7 (3)
**Moderate HD**	8 (5)	13 (7)	5 (3)
**Total**	56 (30)	77 (42)	32 (19)

Breakdown of number of subjects from different subject groups included in each sample set. Number of females in brackets.

Subjects were recruited from the patients and relatives attending the multidisciplinary Huntington’s disease clinic at the National Hospital for Neurology and Neurosurgery, University College London Hospitals NHS Trust; some subjects were also participants in the TrackOn-HD [20] or PADDINGTON studies [21] and had brain imaging available. Informed consent was obtained from all participants, and the study was conducted in accordance with the declaration of Helsinki. Ethical approval to undertake these analyses was provided by the National Research Ethics Service Committee London—Queen Square.

### Biosample collection

8ml whole blood was collected in BD Vacutainer^®^ CPT^™^ Cell Preparation Tubes and processed within two hours according to manufactures guidelines [19]. Briefly, tubes were centrifuged at 1500g for 20 min at 22°C (with no brake) to fractionate the cells using gradient density centrifugation. The cell layer was washed twice with cold phosphate buffered saline before the PBMC pellet was snap frozen on dry ice and transferred to a -80°C freezer until analysis.

### HTT protein MSD electrochemiluminescence assays

ELISA-based MSD electrochemiluminescence immunoassay platforms were used as previously described [16]. Huntingtin levels were measured by four different assays in PBMCs. These four assays, which we have termed A, B, C and D, each used a combination of two antibodies, one capture and one detection (Fig 1).

Detailed description of the antibodies other than pAb139 have been published [16]. Antibody epitope mapping is described with reference to GenBank Accession CAD38447.1; all the antibodies used are raised to human huntingtin. Briefly, pAb137 (CHDI-90000137) is a rabbit polyclonal antibody for HTT amino acids 4–19 (acetyl-LEKLMKAFESLKSFQQC-amide). MW1 is a mouse monoclonal antibody against the expanded polyglutamine domain; Li mapped the epitope to be around 10 glutamines [22]. pAb146 (CHDI-90000146) is a rabbit polyclonal antibodies for HTT amino acids 54–70 (acetyl-QLPQPPPQAQPLLPQPQC-amide) which corresponds to the polyproline region [16]. MAB2166 is a mouse monoclonal antibody whose epitope has been mapped to a 15-amino acid region spanning amino acids 445–459 [23]. pAb139 (CHDI-90000139) is a rabbit polyclonal antibody raised using procedures similar to pAb137 described in Macdonald et al. 2014 against a synthetic peptide corresponding to residues 3109–3134 of the huntingtin protein, specifically acetyl-LDRRAFQSVLEVVAAPGSPYHRLLTC-amide near the C-terminus.

Using combinations of these antibodies, specific areas of the huntingtin protein were targeted (Fig 1). Assay A, which combines MW1 and pAb146 is specific for the polyglutamine-expanded mutant HTT. The other three antibody combinations measure different segments of polyQ-independent HTT. Notably, the N-terminal region assay, B, is polyglutamine-independent. A further amplification step for assays A and B was included by using a biotinylated anti-mouse or anti-rabbit antibody after the MW1 or pAb137 detection antibody incubation respectively, followed by streptavidin-sulfoTAG detection [16].

For every estimated 5 μl pellet size, 100 μl of lysis buffer (0.2% Tween, 0.05% SDS, 0.1 mM EDTA, 1mM PMSF, protease inhibitors in PBS) was added and cells were lysed by vortexing. After clearing the lysate by centrifugation, total protein concentration was measured using a bicinchoninic acid assay (BCA, ThermoScientific) according to standard procedures. A fixed amount of lysate was used for the MSD assay, making up the total volume with lysis buffer.

To allow inter-sample comparison, signal was divided by plate background, then corrected for protein loading volume (30 micrograms in sample set 1, 17 micrograms in set 2). The corrected data were used for subsequent analysis. Standard curves were used to confirm that the samples were within detection range, but because of HTT’s limited stability in solution they were not used to normalise data.

All analyses were performed by operators blinded to subject group and disease stage.

### Imaging

For a subset of subjects in sample set 2 who had undergone 3-Tesla volumetric MRI as a part of another study (TrackOn-HD or PADDINGTON London cohorts), the relationship between rates of caudate and whole brain atrophy and HTT species levels was examined. Atrophy rates provide a quantifiable measure of disease-associated changes in brain volume [20]. Details of imaging protocols and rigorous quality control procedures are provided elsewhere [20, 21]. In brief, follow-up scans were positionally matched to baseline images, and change over 36 months was calculated using the robust boundary shift integral technique [24, 25]. All boundary shift integral changes were annualized before statistical analysis. All image analysis was carried out by an observer blinded to subject identity and group.

The effect of a 0.1 fold increase in HTT signal over background measure on imaging variables was estimated for each assay. Analysis was restricted to 17 subjects for whom imaging was performed within 4 months of biosample collection.

### Statistical analysis

To estimate the reliability of the assays, intra-class correlation (ICC) coefficients were used to examine the proportion of total variance explained by between-subject variability. The larger the coefficient, the less variability within subject relative to the variation between subjects, and so the higher the reliability of the assay. A linear mixed model was fitted to the data at the individual replicate level, and the ICCs estimated (early disease and moderate disease subjects were combined). Standard errors for the 95% confidence intervals were calculated using the delta method [26]. Analyses were adjusted for stage, age and sex and group to remove any between-subject variability attributable to these factors.

Since the HTT measurements were a continuous measure, in order to quantify the associations with disease stage/burden after adjusting for covariates, ordinary least squares (OLS) regression was used to analyse the data. Between-stage differences in HTT levels were analysed adjusted for age and sex. The OLS regression analysis was performed using the mean of the replicates for each protein type as the primary outcome: for the first sample, two readings were averaged; for the second set, three readings were averaged. Linear contrasts, which are linear combinations of parameters used to estimate quantities of interest, were used to determine the difference between disease stage groups.

The disease burden score (DBS) which encapsulates the expected burden of pathology relative to the subject’s age and CAG repeat length [27], and correlates with clinical endpoints [5], is calculated as: DBS = Age × [CAG-35.5]. As for the relationship with disease stage, to evaluate the relationship between DBS and huntingtin species levels, OLS regression was used. In order to fully understand this association it was necessary to consider adjustments for both age and CAG in this model (in addition to sex), to see whether any unadjusted association with DBS is driven simply by age, or CAG, or whether the interaction between the two (DBS) is also important. To have more easily interpretable parameter estimates from this model, we centred age at 50, CAG at 35.5, and entered a centred interaction term between age and CAG (Age − 50)×(CAG − 35.5) in place of disease burden. The parameter estimates and standard errors for age, sex and the interaction term are equivalent to what would have been found had DBS been used in its original form; however this parameterisation gives better accuracy and more useful interpretation of the estimate for CAG.

Cross sectional associations between imaging measures and HTT were similarly analysed using OLS regression with adjustments for age, sex and CAG.

## Results

### The assays used in this study are reliable

Intra-class correlation coefficients (ICC) comparing the duplicate (1^st^ sample set) or triplicate (2^nd^ sample set) samples demonstrated that the reliability of these assays was generally high: ICCs were greater than 0.75 for all assays in the larger second sample set in which we ran the assay three times (S2 Table). For the mHTT assay A in the second sample set the ICC was 0.83, and the within subject standard deviation 1.04.

### Mutant HTT increases with advancing disease stage in two sample sets

**Fig 2:**
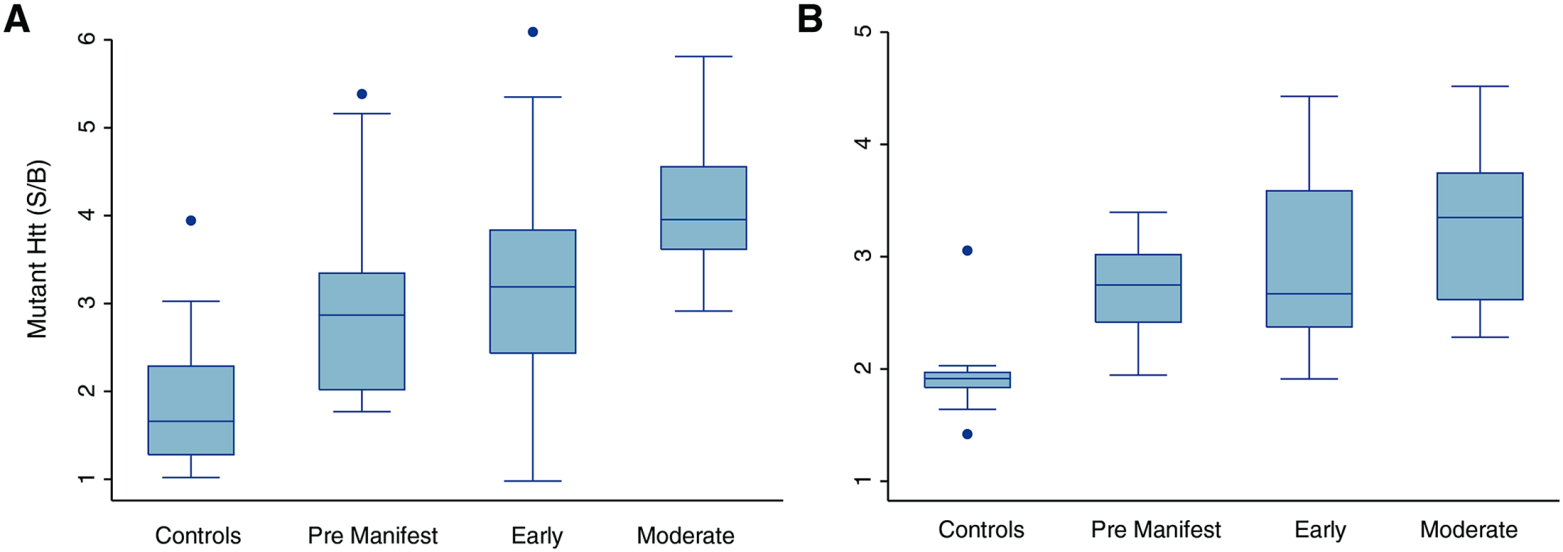
Successful quantification of mHTT in PBMCs from 8ml whole blood in HD. These data confirm that mHTT levels in leukocytes are higher at more advanced disease stages. Assay A (mHTT N-terminus assay) was used to selectively quantify mHTT: S/B, signal over background. A) Sample set 1 (mean of 2 replicates); (B) Sample set 2 (mean of 3 replicates).

Analysis of mHTT protein levels (assay A) across groups in both sample sets (Tables 2 and 3, Fig 2) indicates soluble mHTT is detected at higher levels in mutation carriers compared with controls. We confirm that, even in these heterogeneous cells from a small 8ml blood sample, the mHTT level tends to increase with advancing disease stage, as previously reported [13]. There was a statistically significant linear trend (p<0.001) for this increase in both sample sets. There were significant differences (p < 0.003) in the level of mHTT between all stages of disease and controls (premanifest HD, early HD and moderate HD) in both sample sets (Table 3). There were also significant differences in the level of mHTT between moderate and premanifest HD in both sample sets, however the differences between moderate and early HD only approached significance while early vs premanifest HD were not significant (Table 3).

**Table 2:** Results for each sample set and disease stage.

	Sample set 1		Sample set 2			
Assay (Region)	A Mutant HTT	C HTT Mid region	A Mutant HTT	B HTT N terminus	C HTT Mid region	D HTT C terminus
**Controls**						
n	20	20	23	23	23	23
mean	1.87	7.40	1.93	2.61	4.63	2.22
St. dev.	0.76	3.24	0.28	0.31	1.37	0.35
**Premanifest**						
n	14	14	23	23	23	23
mean	3.03	5.42	2.69	2.56	4.50	2.17
St. dev.	1.11	1.79	0.39	0.39	1.38	0.34
**Early HD**						
n	14	14	18	18	18	18
mean	3.30	6.11	2.97	2.71	5.03	2.13
St. dev.	1.32	2.76	0.77	0.46	1.57	0.28
**Moderate HD**						
n	8	8	13	13	13	13
mean	4.12	7.17	3.29	2.86	5.28	2.40
St. dev.	0.89	1.97	0.75	0.46	1.66	0.49

Unit is assay signal over background. Refer to Fig 1 for details on the assays. (HTT = huntingtin; n = number of samples; St. dev. = standard deviation).

**Table 3:** Comparison of mHTT levels in subjects with different disease stages.

	Mutant HTT (Sample set 1, n = 56)		Mutant HTT (Sample set 2, n = 77)	
	Estimate (95% CI)	P value	Estimate (95% CI)	P value
Pre HD vs Controls	1.14 (0.43, 1.84)	0.002	0.73 (0.41, 1.06)	<0.001
Early HD vs Controls	1.37 (0.63, 2.11)	0.001	0.98 (0.64, 1.32)	<0.001
Moderate HD vs Controls	2.25 (1.3, 3.21)	<0.001	1.34 (0.98, 1.71)	<0.001
Moderate HD vs Pre HD	1.12 (0.07, 2.17)	0.038	0.61 (0.23, 1)	0.002
Moderate HD vs Early HD	0.88 (-0.03, 1.8)	0.058	0.37 (-0.02, 0.75)	0.062
Early vs Pre HD	0.23 (-0.6, 1.06)	0.576	0.25 (-0.11, 0.61)	0.176

Between-stage comparisons of mHTT levels in sample set 1 and 2 using Assay A. Age and sex adjusted. Total number of subjects for sample is indicated, but number of subjects contributing to each comparison depends on the number of subjects in each stage (available in Table 1).

### No robust relationship between poly-Q independent HTT species and disease stage

**Fig 3:**
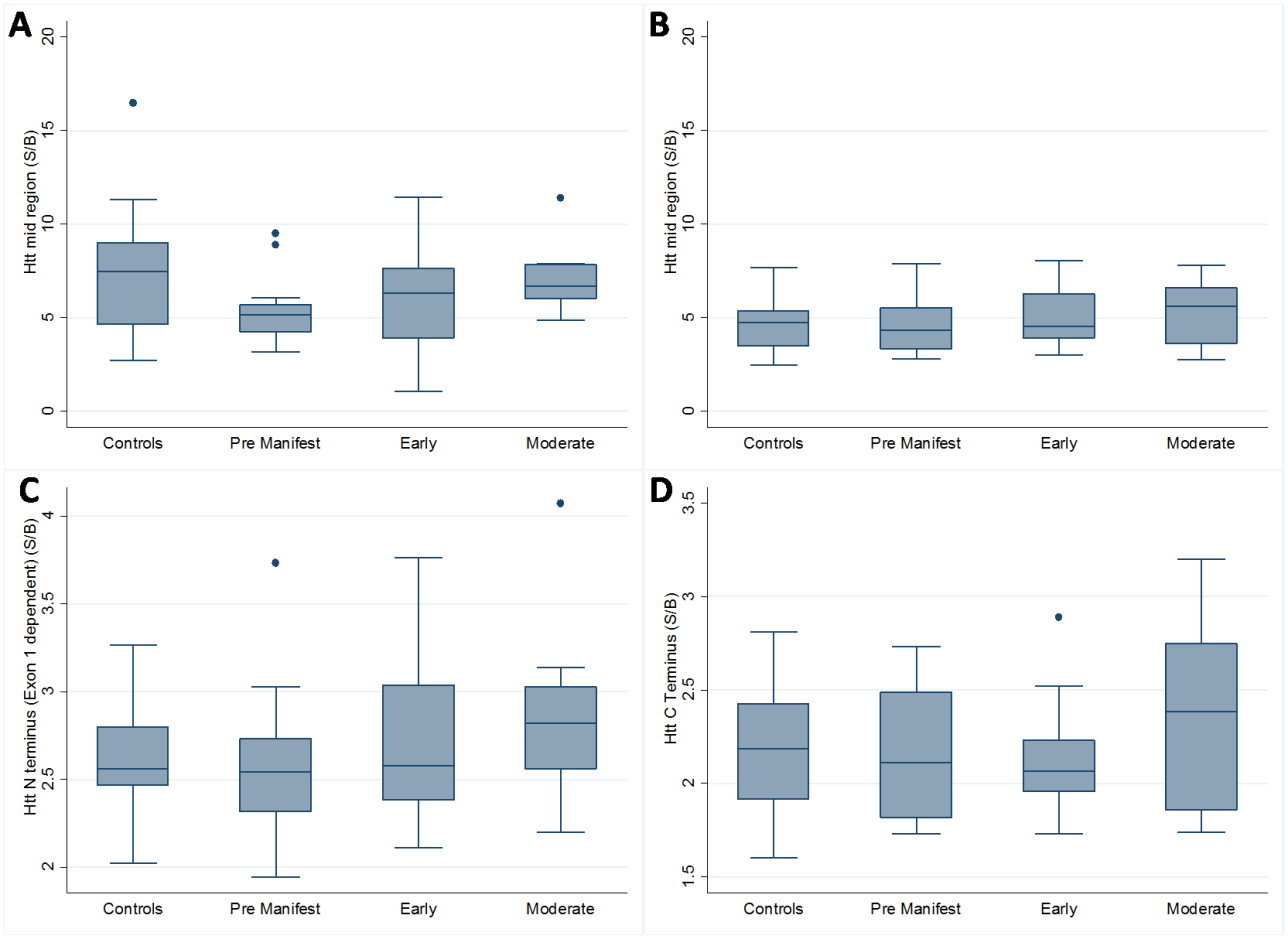
Box plots showing average polyglutamine independent HTT levels across patient subgroups. (S/B: Signal / Background) No significant differences are seen between HTT species levels and disease stage, other than a difference in C-terminal HTT between Moderate and Early HD patients. (A) Distribution of Mid Region HTT (Assay C) in sample group 1. (B) Distribution of Mid Region HTT (Assay C) in sample group 2. (C) Distribution of polyglutamine-independent N terminal HTT (Assay B) in sample group 2. (D) Distribution of C terminal HTT (polyglutamine-independent Assay D) in sample group 2.

Levels of poly-Q independent HTT mid-region species (Assay C) showed no clear trends across groups in either sample set 1 or 2 (Fig 3). In the second sample set N-terminal (Assay B) and C-terminal (Assay D) Huntingtin species were also examined: there was no association with disease stage for either, except for a modest difference in C-terminal HTT between moderate and early stage HD subjects (P = 0.026) (Fig 3).

### Relationship between HTT species and CAG repeat length, disease burden score and sex

**Fig 4:**
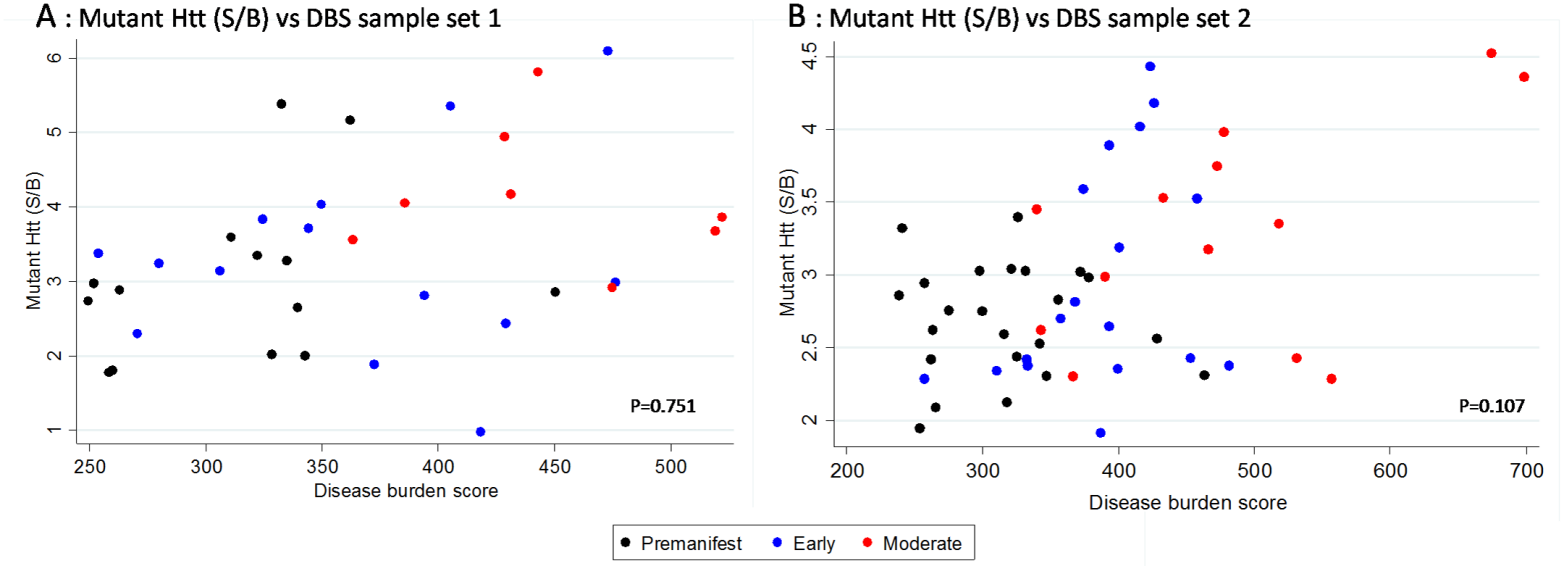
Association between DBS and mHTT (S/B: Signal/Background, mean of replicated samples) in sample set 1 (A) and 2 (B). P values adjusted for age, sex and CAG.

In sample set 2 higher levels of mutant huntingtin were associated with a higher DBS when only disease burden, age and sex were included in the model (p = 0.001). When the CAG repeat was included as a covariate, the relationship with disease burden was no longer significant, but the CAG term was, implying that increased CAG repeat length *per se* is associated with a higher mHTT signal (Fig 4 and S3 Table). In the smaller sample set 1, estimates were consistent with those found in the larger sample, but no statistically significant relationship between mutant huntingtin levels and sex, DBS, age or CAG repeat length was found (Fig 4 and S3 Table).

**Fig 5:**
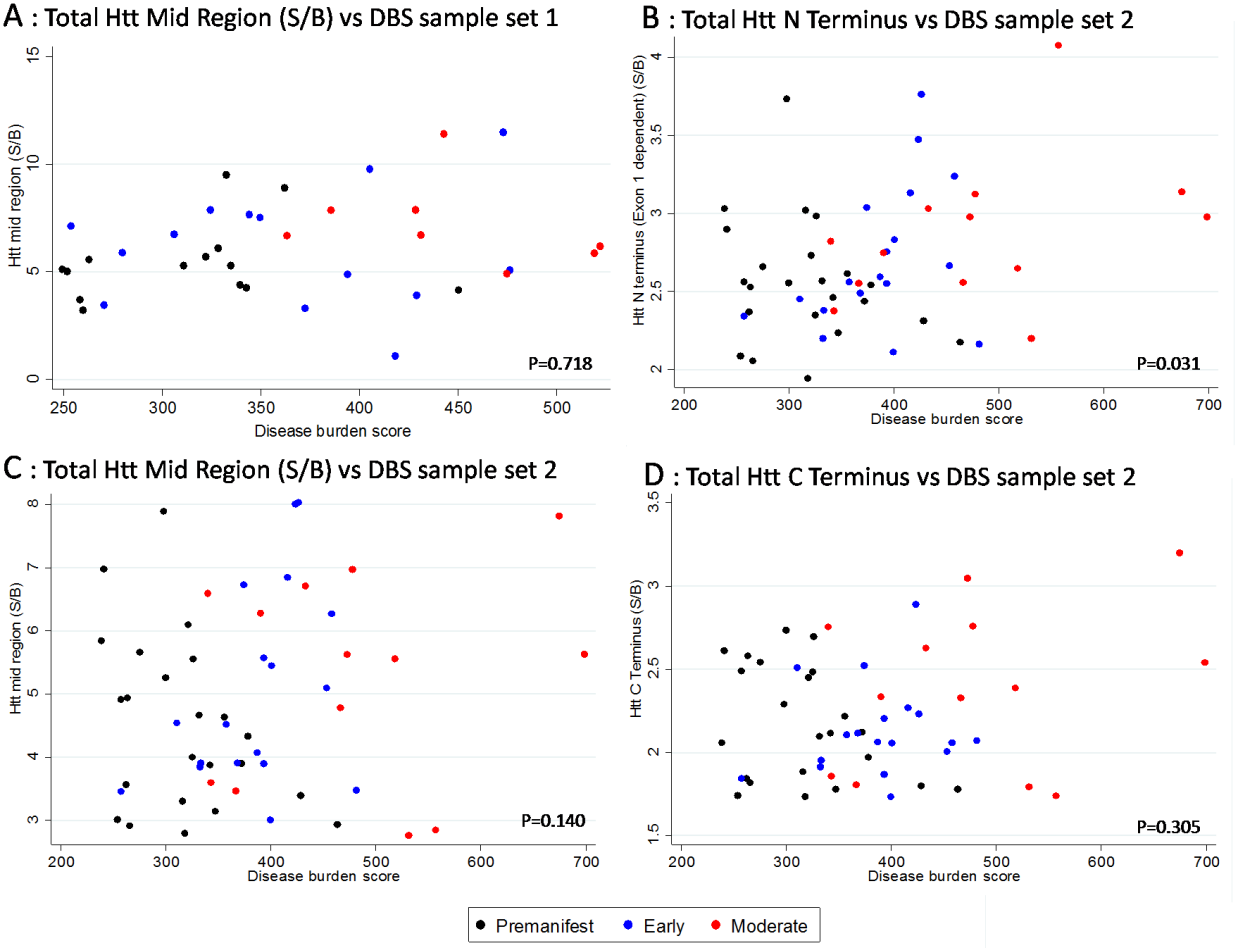
Association between HTT species level (S/B: Signal/Background, mean replicated samples) and disease burden score; adjusted for age, sex and CAG. (A) HTT mid region in sample set 1, (B) N-terminal HTT in sample set 2, (C) HTT mid region in sample set 2, (D) C-terminal HTT in sample set 2). Circles = premanifest, triangles = Early, squares = Moderate.

There were no statistically significant relationships between DBS, CAG, age or sex for either of the HTT mid-region assays (sample sets 1 and 2) (Fig 5 and S4 Table). Similarly there was no relationship between C-terminal HTT and any of these outcome measures. However, for total N-terminal HTT, in the fully adjusted model, DBS was found to be significantly associated with HTT (estimate 0.364, CI 0.035–0.694), as was CAG (estimate of effect of 1 unit increase in CAG on N terminal HTT for subject age 50: 0.089, CI 0.023–0.155) (Fig 4B and S4 Table).

Unexpectedly, we found a significant relationship between sex and mHTT level such that women had a slightly lower level of mHTT than men, in sample set 2 only. This has not been found in previous studies and its significance is unclear, but consequently sex was included as a covariate for all analyses.

### No relationship seen between peripheral huntingtin species and brain imaging measures

Analysis of associations between peripheral HTT levels and (a) the ratio of caudate to whole brain volume as a percentage of the total intracranial volume, expressed as caudate/whole brain volume (%TIV), or (b) caudate/whole brain atrophy rates for the year preceding the sample, showed little evidence of association, though sample size was limited here (S5 Table).

## Discussion

We have demonstrated that the MSD assays used in this study robustly detect mutant and polyQ-independent HTT protein species from human blood with low inter-sample variability: the ICC coefficients for the assays are high. These findings confirm that the previously-described assays [16] are suitable for use in HD disease patient cohorts, enabling the study of HTT species *ex vivo*.

The assays work on small volumes of blood and use unsorted PBMCs generated by a simple processing protocol, meaning that these techniques would be useful for multisite clinical trials and studies. Along with the increased dynamic range of MSD assays compared to other methods of HTT detection, the ability to multiplex assays in a high-throughput manner, and the high throughput on 96- or 384-well format, these assays are suitable for clinical trials in which a peripheral HTT biomarker is important.

We have confirmed our previous finding that higher levels of mHTT are found in PBMCs as HD progresses [13]. The antibody pair pAb146-MW1 specifically detects human HTT protein in a polyglutamine-dependent manner (assay A) [16]. However, we found a background signal when using the MW1 antibody in controls that may be due to off-target background or residual detection of the non-expanded protein by MW1, which recognizes a sequence of approximately 10Qs in the HTT protein [22].

It is interesting that there was no statistically-significant relationship between polyQ independent N-terminal fragments and disease stage in sample set 2. Assay B, the N-terminal protein assay using the pAb146-pAb137 antibody combination, enables the detection of both wild-type and mutant HTT fragments. While the two antibodies are either side of the CAG repeat, the assay is independent of polyglutamine length (Fig 1). Though there was a trend for polyglutamine-dependent N-terminal fragments to increase with disease stage, polyglutamine independent N-terminal fragments, including both expanded and non-expanded alleles did not, suggesting that the observed increase with progression is mHTT-specific. The second sample set also examined C-terminal HTT species and showed there was a significant, but small, effect in C-terminal HTT between moderate and early stage subjects (P = 0.026), the meaning of which is unclear.

The reason for increased mHTT levels in patients with advancing disease stage has not been conclusively established, and putative explanations have been discussed [13]. The assays rely on pairs of antibodies with epitopes at different points on HTT (Fig 1) and they may be detecting either full-length HTT (mutant or wild type), smaller fragments of the protein that encompass the antibody binding sites, or a possible combination of these two. The increase in mHTT signal could reflect an increase in full-length or fragmented N-terminal species of HTT; changes in tertiary/quaternary structure due to folding or complex formation resulting in loss of epitope shielding; or somatic expansion of the CAG tract increasing the number of glutamine residues[28, 29].

Our cell populations were short lived (as in Weiss *et al*,), so mHTT accumulation within post-mitotic cells is unlikely: monocytes typically persist for 2–8 days [30], T-lymphocytes for 11–13 weeks [31] and B-lymphocytes for 10–12 weeks [32], with just a small fraction of lymphocytes persisting as memory cells. Haematopoeitic stem cells in bone marrow are life-long, however, and may accumulate mHTT with ageing. The N-terminal species may be the product of increased HTT production in those with advanced disease or higher disease burden: the CAG expansion may result in aberrantly-spliced isoforms of *HTT* exon1 mRNA that are translated into N-terminal fragments [3, 33]. Additionally, full-length HTT may be proteolytically cleaved to form these N-terminal mHTT fragments [2].

Somatic instability has been implicated in the pathogenesis of HD [34]. While historically somatic expansion was not detected in blood cells [35] it does occur in lymphoblasts [29] and we cannot exclude the possibility that it may be detected in blood using new techniques. Even if somatic instability does occur in blood, however, it is unlikely to be sufficient to account for the observed increase in polyQ dependent binding, since the assays are more dependent upon concentration than polyQ length. By way of illustration, consider that an overall increase from 40 to 50 glutamines might be needed to account for the 33.1% and 22.3% increases we observe between premanifest and manifest in samples sets 1 and 2 respectively. This is because the valency of MW1 is ~10 glutamines [22], so an increase from 40 to 50 glutamines might enable 5 rather than 4 antibodies to bind, giving a 25% increase in signal.

A positive correlation between mHTT and disease burden had previously been demonstrated in monocytes, B cells and T cells [13]; however, that study did not adjust for CAG in its main analysis. Without CAG in the model, we found DBS was associated with mHTT in sample set 2. However this association became non-significant with the addition of CAG. With both CAG and age controlled for in our model, DBS is equivalent to an interaction term between CAG and age. The non-significance of this term means we have found no evidence that the effect of CAG on HTT changes with age, or that the effect of age on HTT changes with CAG. Although non-significant, the estimate was still in the expected direction; the presence of multiple cell populations in our study may increase variance, meaning the present study was not powered to detect a relationship between DBS and mHTT in PBMCs, and suggesting that a larger cohort would be needed to examine this. CAG repeat length was found to be positively associated with mHTT level in the larger sample set 2. Increased length of the polyglutamine expansion is associated with increased MW1 antibody binding [16, 22], but our study includes only a narrow range of repeat sizes (standard deviation 3.40), meaning this effect is unlikely to account for the results. Intriguingly there was a significant relationship between the polyglutamine independent N-terminal fragment of Huntingtin and disease burden in sample set 2, and a significant relationship of small effect with CAG repeat length. Although interesting, it is plausible that this is simply a chance finding, as we explored many associations and did not make any corrections to our significance level for multiple testing.

mHTT was lower in women than men in sample set 2, and there were parallel, though non-significant, relationships between HTT species and sex in all assays. HD phenotypic measures have previously been shown to have complex relationships with sex [36]. However it is also possible that our findings are due to haemodynamic differences between males and females [37, 38].

In summary, we have confirmed the previously reported findings of an increase in soluble mHTT with disease stage in patient blood cells, though for the first time in PBMCs. PBMCs are readily available, making this assay amenable to large-scale clinical studies and trials. The MSD electrochemiluminescence immunoassay platform is sensitive, can be validated for clinical trial use, and is standardisable across sites. We conclude that these assays have potential as a means of understanding the role of different HTT protein species *ex vivo* and warrant further investigation for use in HTT-modulating therapeutic studies.

## Supporting information

S1 Table Demographic data on the cohort examined, divided by sample set. (XLSX)

S2 Table Estimated between and within subject standard deviations and Intra-class correlation coefficient (ICC) with 95% CI for sample sets 1 and 2. (XLSX)

S3 Table Estimated effect (95% CI) and p value of one unit increase in DBS on mutant Htt levels. (XLSX)

S4 Table Estimated effect (95% CI) and p value of one unit increase in DBS on Htt levels. (XLSX)

S5 Table The effect of 0.1 increase in signal/background measure has no significant relationship with brain imaging measures. (XLSX)

S6 Table Raw data used for Figs 2, 3, 4 and 5. For each subject the signal / background (S/B) result is given for each assay performed, these data are used in Fig 2 (mHTT) and Fig 3 (N-terminal, mid region and C-terminal huntingtin species). Age at testing corrected to the nearest half year, CAG repeat length, and Disease burden score (DBS), are provided for each visit; these were used with the S/B data to produce Figs 4 and 5.
(XLSX)
